# A Strand-Specific RNA–Seq Analysis of the Transcriptome of the Typhoid Bacillus *Salmonella* Typhi

**DOI:** 10.1371/journal.pgen.1000569

**Published:** 2009-07-17

**Authors:** Timothy T. Perkins, Robert A. Kingsley, Maria C. Fookes, Paul P. Gardner, Keith D. James, Lu Yu, Samuel A. Assefa, Miao He, Nicholas J. Croucher, Derek J. Pickard, Duncan J. Maskell, Julian Parkhill, Jyoti Choudhary, Nicholas R. Thomson, Gordon Dougan

**Affiliations:** 1 Wellcome Trust Sanger Institute, Wellcome Trust Genome Campus, Hinxton, Cambridge, United Kingdom; 2 Department of Veterinary Medicine, University of Cambridge, Cambridge, United Kingdom; Universidad de Sevilla, Spain

## Abstract

High-density, strand-specific cDNA sequencing (ssRNA–seq) was used to analyze the transcriptome of *Salmonella enterica* serovar Typhi (*S*. Typhi). By mapping sequence data to the entire *S*. Typhi genome, we analyzed the transcriptome in a strand-specific manner and further defined transcribed regions encoded within prophages, pseudogenes, previously un-annotated, and 3′- or 5′-untranslated regions (UTR). An additional 40 novel candidate non-coding RNAs were identified beyond those previously annotated. Proteomic analysis was combined with transcriptome data to confirm and refine the annotation of a number of hpothetical genes. ssRNA–seq was also combined with microarray and proteome analysis to further define the *S*. Typhi OmpR regulon and identify novel OmpR regulated transcripts. Thus, ssRNA–seq provides a novel and powerful approach to the characterization of the bacterial transcriptome.

## Author Summary

We have applied a novel, strand-specific variation of RNA–seq (ssRNA–seq) to an analysis of the prokaryotic enteric pathogen *Salmonella enterica* serovar Typhi, the causative agent of Typhoid fever. Strand-specific data facilitated a high-resolution analysis of RNA transcription at a whole genome level with base-pair resolution. Using this technique, we were able to resolve overlapping transcripts of many genes, identify novel small RNAs, improve the accuracy of annotation, verify operon structure, and identify both transcriptionally active and inactive regions. We have compared the ssRNA–seq approach to standard RT–PCR and microarrays, validating the data. ssRNA–seq was used to redefine the OmpR operon that contributes to the pathogenicity of Typhi, identifying several novel OmpR regulated genes and operons. Finally, we have linked the ssRNA–seq data to the proteome and have provided simple open-access informatics tools to simplify interrogation of the data.

## Introduction

DNA sequencing has been exploited to determine the whole genome sequence of hundreds of prokaryotic and eukaryotic species, facilitating gene identification, transcriptomics and the linkage of genotype to phenotype. To date, genome-wide analysis of the transcriptome has relied to a significant degree on the use of DNA microarrays. However, recent advances in DNA sequencing technologies have facilitated the determination of nucleotide sequence with a genomic read depth several orders of magnitude greater than was previously possible. These novel sequencing approaches have been successfully applied to studies on the transcriptome of eukaryotic genomes [1],[2] and to chromatin immunoprecipitation analysis [3],[4].

Bacterial genomes are relatively small and have a high density of coding sequences (CDS) in comparison to most eukaryotes. For example, the chromosome of *Salmonella enterica* serovar Typhi (*S.* Typhi), the causative agent of typhoid fever, is ∼4.8 Mbp in length, with ∼4,700 genes currently defined in available annotation [5],[6]. *S.* Typhi, unlike most *Salmonella* serotypes that have a broad host range and are associated with localised gastroenteritis, are host adapted (human restricted) and cause systemic infections. The genome of *S.* Typhi harbours many interesting features including horizontally acquired genetic islands specific to this serotype and ∼220 pseudogenes. These pseudogenes are potentially inactivated but many are intact in related host-promiscuous serovars such as *S.* Typhimurium [7]. *S.* Typhi express a polysaccharide, known as the Vi capsule, which is encoded on a large composite element known as *Salmonella* pathogenicity island (SPI)-7 that resembles a conjugative transposon [8]. *S.* Typhi also harbours several putative prophage elements, some of which are absent from *S.* Typhimurium and *E. coli* [9]. Prophages can encode virulence-associated cargo genes, which are not essential for phage viability [9].

In this study we exploit a novel ssRNA-seq method to identify the transcriptional template strand for both coding and non-coding sequences of *S.* Typhi Ty2 at a whole genome level using Illumina-platform high-throughput sequencing. We have identified many putative novel small non-coding RNAs (ncRNAs) and characterised mRNA expressed by pseudogenes. Strand-specific analysis has facilitated the re-annotation of a number of genes and by combining transcriptomic with proteome analyses, we have validated the expression of previously hypothetical genes. Further, we quantify differences in gene transcription in an *ompR* mutant and identify novel regions under control of this virulence-associated locus.

## Results

### Mapping DNA sequence reads generated by Illumina-based ssRNA–seq to the annotated *S.* Typhi Ty2 genome

**Figure 1:**
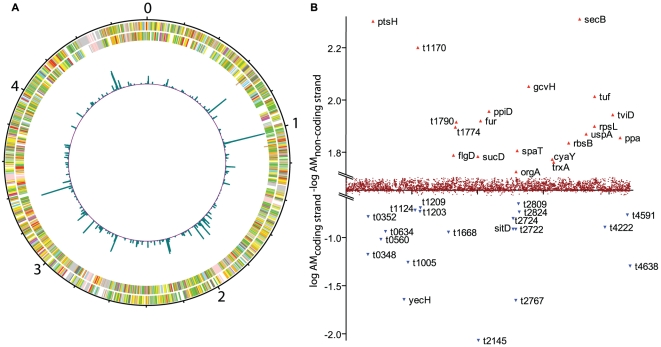
Genome-wide assessment. (A) Circular plot of the reads mapping to the *S.* Typhi Ty2 genome. The outer circle is marked in megabases (0–4). The outermost circles represent CDS on the forward (outermost) and reverse (second outermost) strand coloured according to functional class assigned to CT18 annotation [5], respectively. The inner jagged circle represents a plot of mapped sequence reads with a minimum quality score of 30. Dark shading represents greater (green) than the average and lower (purple). Each base is represented as a pileup of reads and averaged over a window size of 10000 bp. Peaks represent highly sequenced transcripts such as *fliC* (1013788..1015308), *viaB* locus (4494169..4506949) and *sdhCDABsucABCD* (2198361..2208317). (B) Identification of highly expressed genes on the coding and non-coding strands. Log_10_ of AM of the coding strand minus Log_10_ AM of reads mapped to the corresponding reverse strand (y-axis) for each *S.* Typhi Ty2 CDS (x-axis). Greatest and lowest 20 genes are identified by locus tag or gene name.

In order to characterise the *S.* Typhi transcriptome using ssRNA-seq, RNA was prepared from *S.* Typhi Ty2 grown to mid-log phase in LB broth. Since 16S and 23S rRNA was anticipated to be the most abundant RNA species these were depleted prior to sequencing by oligonucleotide hybridisation-mediated selective capture and separation using magnetic beads. The depleted RNA was reverse transcribed to cDNA and sequenced on an Illumina GAI. The resulting 36-base reads were mapped to the *S.* Typhi Ty2 genome. The sequence coverage per base was subsequently plotted and visualised using the genome browser Artemis and DNAplotter, [10],[11] (Figure 1A). To generate the transcript map each base on each strand of the genome was assigned a value derived from the alignment of sequence reads generated from each *S.* Typhi cDNA sample (Table 1). The method employed yields RNA transcript reads in a strand-specific manner and is particularly powerful because it can be used to identify small RNAs and to resolve transcripts originating from overlapping DNA sequences in a manner not possible using low-density microarrays.

**Table 1:** Analysis of ssRNA–seq data mapped to the Ty2 genome.

Flowcell ID/lane	876/2	1104/2	1354/1	876/5	1354/2	1104/3
Strain	BRD948	BRD948	BRD948	BRD948DompR	BRD948DompR	BRD948DompR
Mass of Total RNA (ug)	300	100	100	300	100	100
Total Number of Reads	5608589	7183969	5848604	5375891	3098524	7399877
Reads Mapped	5438270	6513814	5356994	5249193	2774513	6454861
Percentage of Total Mapped to Ty2 (%)	97.0	90.7	91.6	97.6	89.5	87.2
Number of Reads that Mapped Uniquely	1493759	2942477	2326287	1749240	610817	2119151
Percentage of Mapped Uniquely (%)	26.6	41.0	39.8	32.5	19.7	28.6
Reads Mapped to CDS	1235932	2212650	1937241	1500449	491028	1617543
Reads Mapped to NC sequences	257827	729827	389046	248791	119789	501608
Reads mapped to hypothetical genes	131242	266139	264871	248791	78606	275976
GC content (ALL)	0.520	0.418	0.454	0.531	0.496	0.393
GC content (UNIQUE)	0.503	0.443	0.471	0.517	0.479	0.419

**Figure 2:**
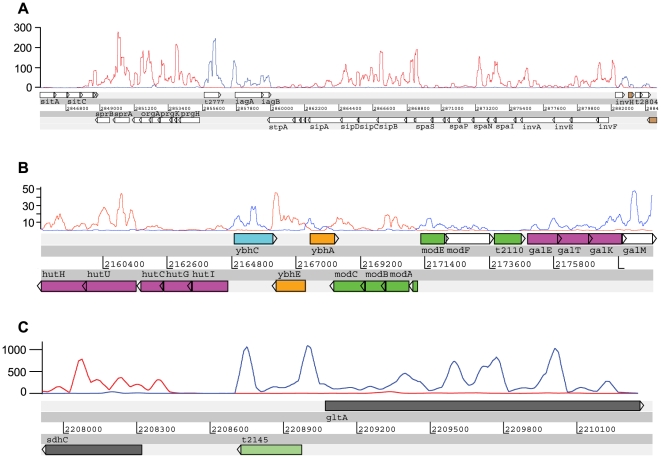
ssRNA–seq data sequence data mapped to the *S.* Typhi Ty2 genome and visualised using Artemis software. (A) *Salmonella* pathogenicity island 1. Sequence data represented as a plot aligned with the annotation after strand specific filtering (annotation represented above or below genes (N.B., not all gene annotations are represented); forward strand blue and reverse strand red, window size = 200 bp). (B) Exemplar genomic region with multiple divergently transcribed genes supports the strand specific mapping of sequence data and previously published annotation. The histidine utilisation operon *hutHUCGI* [62] is transcribed from the reverse strand, followed by three hypothetical genes conserved in *E. coli*. The molybdenum transport system is encoded by two divergently transcribed operons and has been characterised in *E. coli* [63] followed by the galactose operon *galETKM* [64] (forward strand blue and reverse strand red) (window size = 200 bp). (C) An example of a potential mis-annotation. Hypothetical gene t2145 identified as an outlier in Figure 1B exhibits significant sequence reads mapped to opposite strand and upstream region of *gltA*. Forward strand (blue) and reverse strand (red). Window size = 200 bp.

To facilitate analysis of the *S.* Typhi Ty2 transcriptome we calculated the arithmetic mean per base-pair (AM) of mapped sequence reads for the predicted coding strand of the genes currently annotated on the genome and subtracted that of the putative non-coding strand to identify outliers (Figure 1B). Ninety one percent of the reads mapped to the previously annotated *S.* Typhi Ty2 coding strand, providing evidence for successful deconvolution of the nucleotide sequence in a strand-specific manner. This is almost certainly an underestimate of the strand specificity of this method as the remaining 9% of sequences mapped to the unannotated strand were either upstream of a CDS encoded nearby on the opposite strand, putatively identifying riboswitches and promoter regions, or mapped to unannotated or potentially mis-annotated regions. Examples of analyses where strand specific reads were readily identified are shown in Figure 2. As previously reported for ssRNA-seq analysis of eukaryotic RNA [2], the sequence coverage varied across each CDS, indicated by peaks and troughs (Figure 2). However, this profile was remarkably consistent between biological replicates. Importantly, many intergenic regions and 34% of the annotated CDS had few (AM<1) or no mapped reads. For sequence data mapped to a region where CDS orientation is highly “mosaic” the plots align predominantly to the predicted annotation (Figure 2B), further illustrating the strand-specific nature of the ssRNA-seq data. Sequence reads that mapped to non-coding strands may represent transcriptionally active but previously unannotated features of the genome. Indeed, these data enabled us to identify putative errors in the annotation of some genes, including many genes annotated as hypothetical, such as the locus t2145 (Figure 2C). A large amount of sequence data mapped to the opposite strand from this annotated CDS (Figure 2C), which suggests this may not be a hypothetical gene at all but instead the 5′ region, and a putative novel *cis*-regulatory element, of the *gltA* (t2146) gene [5],[6]. In support of this hypothesis, the intact reading frame for this predicted gene is not conserved outside the *Salmonella* but the DNA sequence itself is conserved in this location in many enteric bacteria, including *Escherichia*, *Klebsiella* and *Enterobacter*.

**Figure 3:**
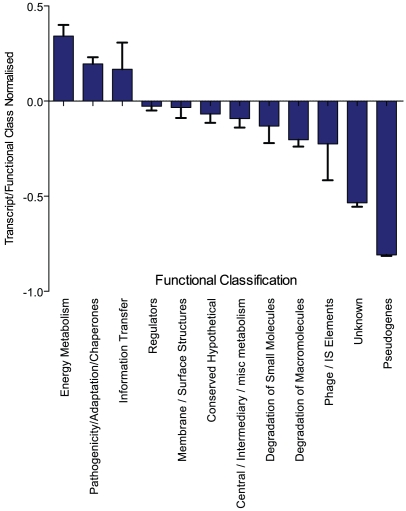
Overview of the *S.* Typhi Ty2 transcriptome generated by ssRNA–seq according to functional classification. The total number of reads/bp mapped to each CDS are assigned to functional class described previously [5]. These data were then normalised by the number of CDS for each function encoded within the entire genome. A ratio of 1 represents transcription of functional class on par with its genome content. A ratio of more than one represents a transcriptionally over-active class, and less than one, under-active.

In order to provide an overview of the gene classes active in the genome-wide transcriptome, we determined the AM for each CDS and by using the previously assigned functional group classification [5] we assigned the number of sequence reads to each of the 12 functional classifications, which were then normalised, relative to total genome content of each class (Figure 3). A ratio of >1 represents a more highly transcriptionally active class. The ratios for outer membrane/surface structures, regulators, conserved hypotheticals and central intermediary metabolism were ∼1. Interestingly, transcriptional reads for CDSs associated with energy metabolism, information transfer and pathogenicity/adapation/chaperones were over-represented in the transcriptome with the ratio ranging from 1.57 to 2.23. As may be expected, transcriptionally silent prophage elements (ratio ∼0.75) are under represented with most of this transcript mapping to putative phage cargo genes (discussed later). Genes predicted to encode proteins required for degradation of both macromolecules and small molecules are under-represented in the reads, which is consistent with these genes being under tight transcriptional control in rich media. Interestingly, although pseudogenes represent 4.6% of the predicted CDS of *S.* Typhi Ty2, only 0.69% (ratio ∼0.15) of the entire ssRNA-seq generated transcriptome mapped to these genes, indicating a 10-fold reduction in expected transcriptional activity.

### ssRNA–seq and proteome analysis of *S.* Typhi hypothetical genes

**Figure 4:**
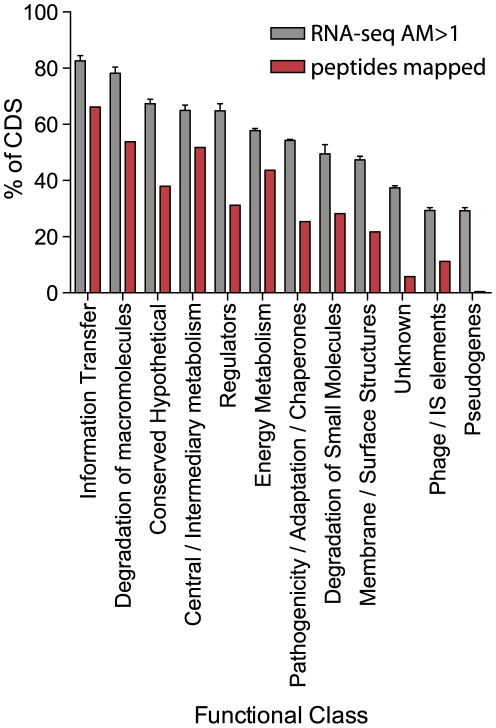
Overview of *S.* Typhi Ty2 transcriptome and proteome assigned to functional class. The percentage of CDS in each functional class with an AM≥1 (grey bar). The percentage of hypothetical CDS with at least one mapped sequenced peptide (FDR<0.076) (red).

Many annotated predicted genes of the published *S.* Typhi genomes were assigned in the absence of clear protein homologies or direct evidence for transcription or translation into a protein product. We therefore carried out a comprehensive survey of transcript sequence and proteomic analysis of predicted genes in *S.* Typhi Ty2. We determined the AM sequence coverage and mapped peptides identified from fractions of *S.* Typhi Ty2 using LC-MS, for each predicted gene in the available annotation. The proportion of predicted genes in each functional class with AM sequence coverage >1 and those with at least one mapped peptide from proteomic analysis were determined (Figure 4, Table S1). The groups with the greatest proportion of transcriptionally active genes were those of information transfer (transcription and translation) and degradation of macromolecules, as would be expected for actively dividing bacteria in mid log phase. Pseudogenes, phage/IS elements, and ‘unknown’ genes were the least transcriptionally active and few peptides were mapped to products of genes from these classes. Relatively few pseudogenes had significant transcription and very few peptides mapped to these interrupted genes, suggesting that following pseudogene formation, transcription and translation are rapidly lost. It is also evident that functionally unassigned predicted hypothetical genes are frequently transcriptionally inactive with few mapped peptides. This may be because these are genes are only activated by specific environmental signals such as *in vivo* within host tissues or they are not true CDS and represent mis-annotation.

**Figure 5:**
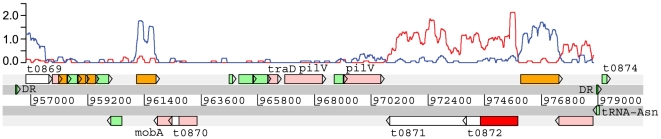
Annotation of a variable region of *S.* Typhi Ty2. Alignment of ssRNA–seq sequence data to an unannotated region of Ty2 provided evidence for putative CDS. This region is encoded between annotated genes t0869 and t0874. It may have been missed in the published annotation due to the variation between Ty2 and the previously sequenced *S.* Typhi CT18 strain [5]. Resequencing of 21 *S.* Typhi isolates identified 2 different configurations of this variable region [12] and were annotated as ST20a and b. This figure represents ST20b. Colour code: white - previously annotated genes; pale green - hypothetical genes; orange - conserved hypothetical genes; pink - genes involved in integration or mobilisation; red - DNA modification genes. DR; direct repeats. Transcript represented by plot (log scale, forward strand (blue) and reverse strand (red), window size 200 bp). Note that the previously annotated CDS t0872 (white) represents only the 3′ end of the gene. The remaining segment of the gene is indicated (red).

We also identified a variable [12] and previously unannotated region of the *S.* Typhi Ty2 genome to which mapped a number of transcriptome sequence reads (Figure 5). In this region, transcript data identified six transcribed CDS allowing us to refine the annotation of this region. These highly transcribed genes encode (from left to right) a protein with similarity to hypothetical proteins from a number of sequenced enteric bacteria (previously annotated as t0869); a protein with similarity only to a single protein from a marine *Bacillus* sp NRRL B-14911; two proteins with similarity to a restriction endonuclease/methylase pair (t0872 and t0871); and a conserved hypothetical protein with a broad phylogenetic range of matches. The last transcribed gene in this region is a putative phage integrase, which is adjacent to a conserved tRNA-Asn; the whole region is bounded by a 24 bp direct repeat of the 3′ end of the tRNA gene, indicating that this region is likely to be capable of independent integration and excision. Also encoded in this region are two duplicated type-IV pilV-like proteins, MobA-family and TraD-family conjugal transfer proteins, and a number of other genes of unknown function that show little evidence for transcription under these conditions.

### Non-coding (nc) RNA sequences

**Figure 6:**
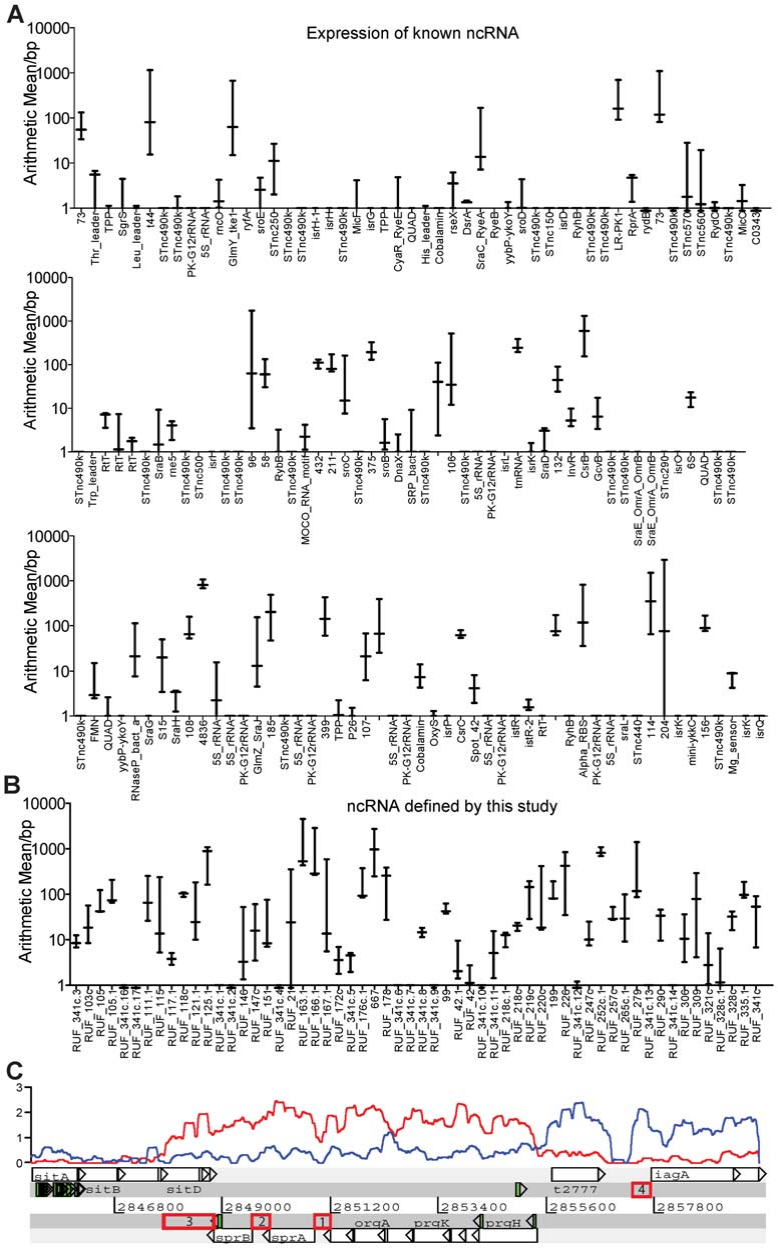
Previously identified and novel putative ncRNA. The AM for each intergenic feature (mean and range) derived over three biological replicates. (A) Previously identified ncRNA and (B) ncRNA elements identified by this study. (C) Putative ncRNA elements in SPI-1. Identification of 4 intergenic regions of sequenced transcript, 3 predicted to be *cis*-acting 5′ elements (upstream of *sprA* (RUF_220c, 1) *sprB* (RUF_219c, 2) *iagA* (RUF_221, 4), and 1 possible 3′ UTR (downstream of *sprB*, RUF_218c, 3), within the cell invasion locus, SPI-1. Transcript represented by plot (predicted ncRNA represented by red box, log scale, forward strand (blue) and reverse strand (red), window size 200 bp).

Forty-two of the 82 ncRNAs annotated by Rfam in *S.* Typhi Ty2 generated transcripts that were detectable by ssRNA-seq analysis, with a range of AM between 1.2 and 438.18 reads/bp (Figure 6A). In addition, many sequence-reads mapped to novel intergenic regions of the *S.* Typhi Ty2 genome that were not previously annotated. Further analysis of such reads has allowed us to annotate an additional 55 genomic features as small ncRNAs based on these data. Many of these novel ncRNAs were not unique to *S.* Typhi Ty2, since putative homologues were identified in other *S. enterica* serovars and other bacterial species (Table S2). We also identified 25 CDS that were preceded by putative 5′ UTR transcripts, 13 of which were more than 150 bp in length (Table S2), as well as 5 novel putative 3′ UTR, 2 of which are adjacent to *sprB* and *ramA*. Subsequently, we determined the AM for each predicted ncRNA (Figure 6B, Figure S1A, S1B), showing that 91 of the 245 elements had an average AM>1. Taken together, this sequence data suggests that there may be many previously unidentified functional ncRNAs present in *S.* Typhi, that are conserved in other bacteria.

Recently a survey of Hfq bound ncRNAs was reported [4]. We mapped 52 ncRNAs identified in this study to *S.* Typhi Ty2 using annotation from the Rfam database [13]. Five of these had an average AM>1, the minimum of which was 1.75 (istR-2) and the maximum 13.38 (STnc250). The lack of overlap between these two datasets may be due to different experimental strategies for isolating and enriching RNA species. The RNA isolation method used in this study was optimised to remove contaminating proteins and may therefore remove RNA species bound to Hfq. Furthermore, the RNA preparation includes a rRNA depletion step, Hfq is known to bind sRNA to mRNA to regulate ribosomal initiation. This has been reported to occur both pre- [14] and post-ribosome initiation [15]. It is therefore possible that HFQ-bound sRNAs are being removed, prior to sequencing.

Transcription and translation in prokaryotes are commonly regulated by changes to the conformational structure of *cis*-acting ncRNAs called riboswitches. These RNAs generally bind metabolites related to the function of their associated downstream genes [16],[17],[18],[19],[20] and have been identified using bioinformatics methods based on sequence conservation of the 5′ UTR. Several known riboswitches, such as *btuB* [17],[21] and TPP [19] were highly represented in the ssRNA-seq data (Table S2). To analyse the putative ncRNAs for the potential to act as putative cis-regulatory elements we used a combination of ssRNA-seq, sequence conservation, secondary structure conservation and further homology search using covariance models. Subsequently, RNAz was used to rank candidates. One of the most interesting regions encoding putative novel ncRNAs was *Salmonella* pathogenicity island 1 (SPI-1) [22] (Figure 4C). Two of these SPI-1 associated transcripts were identified [23] as candidate riboswitches, here designated RUF_220c (1) and RUF_219c (2) (Figure S2A, S2B). RUF_220c and RUF_219c are located directly upstream of the *araC*-like regulators *sprA* (t2988) and *sprB* (t2987), respectively. A third candidate element, which is predicted to be a 3′UTR designated RUF218c (3), is encoded on the antisense strand of the *sitD* gene, an iron transport protein [24], and a hypothetical protein O30622 (t2767), which may have been acquired independently of the rest of SPI-1 [24]. The sequence of RUF_218c is conserved across cyanobacteria, firmicutes and proteobacteria. The *sitA* gene maps sequenced transcripts (average AM = 1.27) and 7 uniquely mapping peptide hits, whereas *sitB*, *sitC* and *sitD* have slightly lower levels of expression (AM = 0.37, 0.38 and 0.61, respectively) and map no sequenced peptides in our proteome preparations. It is possible that RUF218c is an antisense repressor of these proteins, as it is predicted to form a moderately stable minimum free energy secondary structure compared to a shuffled ensemble of sequences that have the same di-nucleotide composition (p = 0.0090). The fourth candidate element, named RUF_221 (4), maps to the 5′ UTR of *iagA* (t2999) (Figure 6C), an invasion regulator [25]. The predicted structure of this RNA (34% G+C) is not supported by other analyses (RNAz probability of 0.0037 and shuffling p value = 0.2627) (Figure S1C). There was also high sequence coverage in the 5′ UTR of t3658 (STY3917 in CT18), an orthologue of *glmS of* E. coli, a glutamine fructose-6-phosphate amino transferase. In certain Gram-positive micro-organisms a riboswitch has been characterised in the 5′ UTR of the *glmS* gene, that also encodes a glutamine fructose-6-phosphate amino transferase [26], suggesting that a candidate *cis*-regulating element is also present in *S.* Typhi.

A further previously unidentified putative non-coding feature is RUF_107c (complement strand, base range 101116..101223), which is highly expressed in these *S.* Typhi Ty2 samples. This element, predicted to be highly structured by RNAz (p = 0.9396), has approximately 115 paralogues in *Salmonella* (Figure S1A). Further, it is conserved across ∼82 bacterial species but is chiefly restricted to Enterobacteriaceae. The genomic context of RUF_107c and its paralogues is not consistent with a *cis*-regulatory or a transposable element, as the sequence does not consistently co-occur with either CDSs or near transposases, respectively.

### Integrated prophage and other putative mobile genetic elements

**Figure 7:**
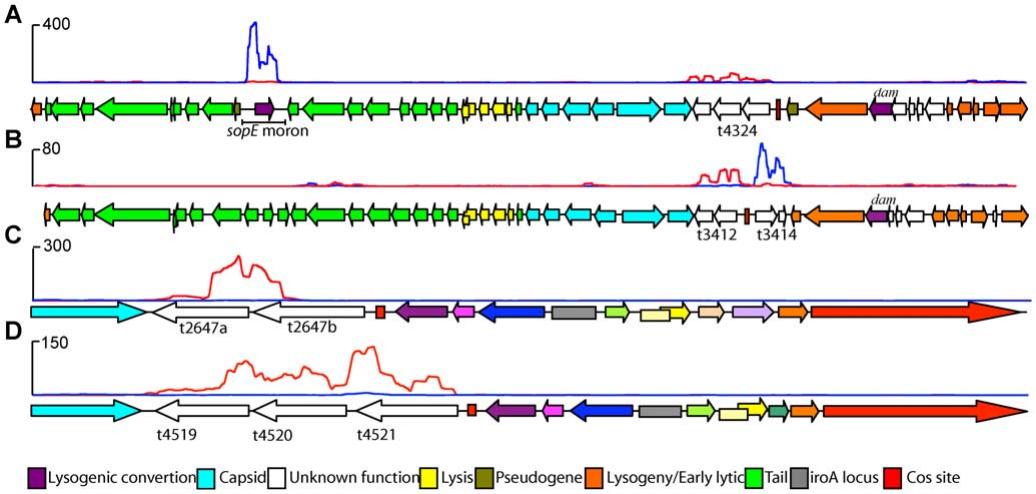
Transcriptionally active prophage genes. (A) Genetic organisation of the SopE prophage aligned with mapped sequence reads illustrates “expression” of the *sopE* moron (AM = 283) and another putative cargo region (t4323–t4325, AM = 21.4, 40.3, 18.6, respectively). Transcription of the *cI* repressor is required for maintaining lysogeny and this region mapped an AM = 5.15 compared with median AM for entire phage = 0.93. (B) Genetic organisation of the ST35 prophage. The low GC region maps significant sequence coverage compared with the prophage “machinery”, putatively identifying it as cargo. Putative prophage cargo in (C) ST2-27 and (D) ST46 with transcriptionally active low GC regions. (All plots, forward strand blue and reverse strand red, window size = 200 bp).

*S.* Typhi harbours a number of distinct prophage, whose complement can vary between the different evolutionary lineages [9],[12]. Such prophages are regarded as being predominantly transcriptionally silent in the genome and can harbour horizontally acquired ‘cargo’ genes potentially encoding factors that modify the virulence of the host bacteria [27],[28]. Our analysis confirms that most of the resident prophage are indeed predominantly transcriptionally inactive (Figure 7) but it is worth noting that the ssRNA-seq mapping was sufficiently sensitive to highlight low level transcription across phage regions involved in maintaining lysogeny (Figure 7). However, we noted that four of the prophages did harbour transcriptionally active regions and that some of these mapped over well-known cargo genes such as *sopE* encoded by the SopE phage (Figure 7A). Cargo genes are non-essential for phage proliferation but may confer fitness to the lysogenised host bacterium [29],[30],[31]. Similar analysis of this prophage and others within the *S.* Typhi Ty2 genome highlights several transcriptionally active regions, which may encode novel cargo genes. Bioinformatics analysis of these regions, in some cases, supports this hypothesis, in that the genes do not encode known phage proteins and have differing GC content than other *S.* Typhi genes [9]. The SopE prophage expresses another region distinct from *sopE* that could encode three putative cargo genes, which are similar to hypothetical proteins found in *Vibrio cholerae* (Figure 7A, t4323, t4324 and t4325). Database searches using the transcriptionally active regions in the ST35 prophage (base range 3500845..3536809) reveal sequence similarity to hypothetical genes found in *E. coli* O157 (Figure 7B genes t3414, t3415). The ST46 prophage (4666742..4677430) encodes three transcriptionally active genes; two have sequence similarity to protein kinases and the third is a candidate threonine/serine kinase (Figure 7D, t4519, t4520, t4521). Thus, these methodologies may provide a novel approach to identifying phage cargo genes expressed during the lysogenic phase. A total of 73 peptides mapped to the four prophages (Figure 7, Table S4) and 59 (81%) mapped to highly transcribed regions containing known or putative cargo genes. Of these remaining, 5 peptides mapped to the highly transcribed cI repressor gene required for maintenance of lysogeny.

### Pseudogenes

*S.* Typhi, in common with other host-adapted pathogens, harbours a large number (∼220) of putatively inactivated pseudogenes [5],[6],[12]. Genome degradation may contribute to host restriction by inactivating pathways essential for infections in the non-permissive host. Theoretically, putative pseudogenes can still express a functional truncated protein domain, as for example has been demonstrated for truncated cytotoxin in *Chlamydia trachomatis* [32],[33]. Based on comparative sequence analysis of 21 *S.* Typhi and two *S.* Paratyphi A genome sequences, the 220 pseudogenes of *S.* Typhi strain Ty2 have been assigned to four groups based on their predicted relative age [34]. We were able to identify nine pseudogenes in *S.* Typhi Ty2 that exhibited high levels of transcription, a property that was independent of their predicted age (Figure S3), suggesting that transcription may be being maintained to express functional domains as RNA or peptides. We did identify one pseudogene, *hdsM* (t4575) with sequenced peptide data, mapping to the open reading frame upstream of the inactivating stop codon. This represents the only significant evidence in this study of peptides mapping to putative pseudogenes. This, combined with the relative lack of transcription from other *S.* Typhi pseudogenes, supports the current interpretation that the majority of these genes are no longer active.

### ssRNA–seq and microarray analysis of a *S.* Typhi *ompR* mutant

The global regulator OmpR is known to regulate the levels of transcription from a number of distinct loci within the *S.* Typhi genome, including the *viaB* locus [35] associated with Vi capsule production and the outer membrane porins *ompC* and *ompS* [36],[37],[38]. OmpR is also known to interact with the endogenous two-component regulator of SPI-2, *ssrAB* in *S.* Typhimurium [39],[40]. The complete OmpR regulon in *S.* Typhi has not been fully defined. We therefore prepared RNA from *S.* Typhi Ty2 and an otherwise isogenic *S.* Typhi Ty2 *ompR* mutant growing simultaneously in LB broth to mid-log phase (OD = 0.6). This RNA was then subjected to ssRNA-seq analysis and supporting conventional microarray analysis as control (see Methods).

To perform a quantitative ssRNA-seq comparison between sequenced products from the *S.* Typhi and *ompR* mutant RNA pools the AM was determined for all CDSs and, for the purposes of this analysis, these values were treated as intensity values similar to those derived by microarray scanning. We did not compare expression of ncRNA in this analysis. Using the AM and the LIMMA package for microarray analysis the data were quantile normalized [41]. Prior to Benjamini-Hochberg false discovery rate estimation and correction (BH-FDR), 305 genes had significantly different levels of sequenced transcript (2-fold change and p-value<0.05) (Figure S4) in *S.* Typhi Ty2 compared with *ompR* mutant derivative. Following application of BH-FDR, differences in sequence transcript was significant in fifteen of these genes (2-fold, adj p<0.05), all exhibiting a significant decrease in transcription in the *ompR* mutant. Consistent with previous reports, the entire *viaB* locus including *tviABCDE*, *vexABCDE* is represented in these 15 genes, [35] as well as *envZ*, the sensing component of the OmpR regulon. The four remaining genes were *slsA* (t3757), *hyaA* (t1458) and hypothetical genes t1459 and t1641 that are discussed below. Importantly, we confirmed that these genes were differentially expressed in the *ompR* mutant by a further method, quantitative PCR assays (data not shown).

Many of the 305 genes with significantly different transcript levels in the *ompR* mutant before BH-FDR correction, such as *ssrAB*, *ompC* and *ompS*, were reported previously to be OmpR regulated in *Salmonella* [36],[38],[39]. Furthermore, 71 of these genes appear to be encoded as 28 separate operons with similar differential expression patterns (Table S4). Indeed, some of these clusters of genes including *ttrRS*, *ssrAB*, *cheBY*, *narZY*, *flgMN*, *flgHIJ*, *modAB*, *phnUV*, *hycGH*, *rplEXN*, *rplFR*, *aceBAK*, have been previously confirmed as operons. This suggests ssRNA-seq identified blocks of differentially transcribed genes increasing our confidence in these findings despite exclusion following application of BH-FDR. Other examples of genes identified by ssRNA-seq include *ssrA* and *ssrB* (expression ratios of 0.09 and 0.31 respectively) known regulators of SPI-2 previously reported to be influenced by OmpR [39],[40]. Furthermore, the flagella genes *fliH*, *fliI*, *fliM*, with expression ratios of 2.08, 2.49 and 2.26 respectively, were in the original list of 305 genes.

Since ssRNA-seq is a new approach to mRNA expression analysis we performed independently a classic microarray analysis on the mRMA prepared from wild type *S.* Typhi Ty2 and *ompR* mutant derivatives as described in Methods and compared the data. We confirmed similar differential expression of 38 of the original 305 genes (two-fold, p<0.05) identified by ssRNA-seq independently by this DNA microarray expression analysis (Figure S4, Table S3). Of the 17 genes of this type that were decreased in expression in both experiments, ssRNA-seq reported a greater difference (ompR/WT) in the expression levels of these apparently down-regulated transcripts compared with microarrays (Figure S4). Genes previously characterised as OmpR-regulated with decreased levels of expression in *S.* Typhi Ty2 *ompR* mutants were *tviABCDE*, *vexABCDE*, *ompC* and *ompS*. Genes not previously described in the OmpR regulon identified in these data included the *slsA* (t3757) gene that is encoded within SPI-3 (also confirmed by direct RT-PCR), that is conserved throughout *Salmonella* and a putative inner membrane associated isochorismatase hydrolase. Isochorismatase hydrolase has been characterised in the phenazine biosynthesis pathway in *Pseudomonas aeruginosa*, potentially involved in antimicrobial activity and induced neutrophil cell death [42]. The hydrogenase uptake gene, *hyaA2* (t1458) is also under represented in the *S.* Typhi Ty2 *ompR* ssRNA-seq data, the microarray data and direct RT-PCR assays. *Salmonella* encodes three predicted hydrogenase operons, two hydrogenase 1 operons (*hyaACDEFt1048* and *hyaA2B2C2D2E2F2t1454*) and a hydrogenase 2 operon (*hybOABCDEFG*) that are important factors in respiration. Interestingly, two subunits of each operon, *hyaA* and *hyaB2*, are pseudogenes in *S.* Typhi Ty2 and CT18 [5],[6]. All three of these operons contribute to virulence in the *S.* Typhimurium murine model [43]. Furthermore, expression of the gene divergently transcribed from *hyaA2*, a putative secreted choloylglycine hydrolase (t1459) is also significantly decreased. The family of choloylglycine hydrolases cleave carbon-nitrogen bonds, exclusive of peptide bonds, and include conjugated bile acid hydrolase and penicillin acylase [44].

Intriguingly, 21 genes were increased in expression by the loss of *ompR* as determined by both ssRNA-seq and microarray analysis. Two contiguous flagellin regulatory genes, *flgN* (t1749) and *flgM* (t1748) were increased in expression in the *ompR* mutant. FlgM is a negative regulator of flagella biosynthesis and a mutation in this gene attenuates virulence in *S.* Typhimurium [45]. FlgN is required for the efficient initiation of filament assembly [46]. The glyoxylate shunt genes (*aceBAK*) are also increased in expression in *S.* Typhi Ty2 *ompR* (confirmed for *aceA* by RT-PCR) and fatty acid catabolism by isocitrate lyase is crucial for macrophage persistence in *Mycobacterium tuberculosis* [47]. Three genes *t3544*, *t3543* and *t3538* that are predicted components of a ribose/arabinose transport operon were also increased in expression. Furthermore, predicted genes *t1788*-*90* were greatly increased in expression in the *ompR* mutant. These genes are contiguous and encode proteins with sequence similarity to a sialic acid transporter, a secreted protein and a sialic acid lyase respectively and are not present in *E. coli*. Molybdate transport is a crucial co-factor for anaerobic metabolism and transcription from two genes, *modAB*, required for its transport were increased in the *ompR* mutant. The levels of transcription of *dppA*, *cstA*, *ybeJ*, *ybfM*, *glnH* and t1709 were also increased in *S.* Typhi Ty2 *ompR* and these encode proteins annotated as periplasmic dipeptide transporter, carbon starvation response, glutamate transport, putative outer membrane, glutamine transport and a hypothetical protein, respectively.

## Discussion

We show here that Illumina-based ssRNA-seq sequencing technology allows the analysis of the transcriptome of the bacterial pathogen *S.* Typhi at the whole genome level and in a strand-specific manner. This technology therefore provides a powerful new approach to studies on bacterial gene expression, pathogenicity and mechanisms involving gene regulation at the level of transcription. By converting RNA to DNA it is possible to profile expression at a genome-wide level in such detail that even subtle features such as regulatory RNA features and small RNA sequences can be readily identified. Indeed, we were readily able to identify known attenuators and similar features in front of the Threonine (*thr*), Tryptophan *(trp)* and other operons (Figure S5). The depth of sequence analysis is sufficient to differentiate levels of expression, facilitating studies on bacteria or their mutant derivatives growing in different environments or conditions. Visualisation and interpretation of the transcript map was simplified by the exploitation of powerful bioinformatics mapping software and a modified version of the genome browsing tool Artemis [48]. Further, the transcriptome analysis was linked to the proteome, providing validation for a number of previously hypothetical genes. Indeed, the analysis was a useful tool for improving the annotation of the genome, redefining the limits of genes and transcripts and identifying novel small CDSs. Our analysis confirmed the expression of many known riboswitches that have recently been characterised and identified many more candidates. Indeed, we have mapped significant sequence data to the 5′ UTR of over 127 genes using ssRNA-seq. Many of the currently annotated riboswitches were predicted bioinformatically and their functionality was previously assessed through *in vitro* phenotyping assays [16],[17],[19]. Our genome-wide survey predicts such elements on a whole genome level providing candidates for further biological analysis. Three of these candidate regions were located in SPI-1, where they may impact on the expression of virulence genes.

Pseudogenes have contributed to apparent genome degradation in a number of host-adapted pathogens. Pseudogenes harbour potentially inactivating mutations that are normally identified through genome annotation programmes. However, the exact mechanisms by which pseudogenes impact on *Salmonella* pathogenesis is not fully understood but is believed to involve a loss of pathways that diversify the mechanisms the pathogen uses to survive in different hosts and tissues [49]. In this report, we demonstrate that the transcription of many pseudogenes is low or absent in a manner that is independent of the predicted age of pseudogene acquisition. However, we did identify several pseudogenes that are transcribed in rich media and peptides mapped to one of these. However, overall the evidence supports the concept that most *S.* Typhi pseudogenes are indeed null mutations.

Analysis of the prophage like elements encoded within the *S.* Typhi genome demonstrates that these are largely transcriptionally silent regions. Even so, the analysis was sensitive enough to identify genes that contribute to maintenance of the prophage state such as repressors of lysogeny. However, using ssRNA-seq analysis we are able to highlight transcriptionally active regions within largely inactive prophage elements. Some of these correlated with previously characterised cargo genes such as *sopE* that can contribute to pathogenicity. We postulate that other transcriptionally active regions within these prophage elements may be novel “cargo genes”. Peptides were mapped back to some of these regions.

Finally, we believe that the approaches described here are potentially applicable to any bacterium and provide a simple route towards the analysis of gene expression. The method, as outlined, has the advantage of providing strand-specific analysis allowing high resolution transcription maps to be generated. The method described is generic in that it can be performed with relatively minimal manipulation of nucleic acid and all the bioinformatics tools described are freely available. Further work will be required to optimise the use of ssRNA-seq for routinely analysing transcription in bacteria. For example, we do not yet know how accurate the quantitative analysis will be at a genome level in different bacteria. Our comparisons between ssRNA-seq and DNA microarray analysis for comparing differential gene expression using *S.* Typhi wild-type and *ompR* mutant derivatives indicates that the two approaches may be complementary but that they may not yield completely overlapping data. Indeed, previous work has shown that different microarray platforms are subject to considerable variability in reported transcription [50],[51]. Nevertheless, by combining both approaches we have identified sets of both known and novel OmpR regulated genes.

## Materials and Methods

### Bacterial strains, growth, and RNA preparation

The bacteria used were all derivatives of *S.* Typhi Ty2 [6]. The *ompR* null mutant was made in the *S.* Typhi Ty2 by the red recombinase method [52] using the kanamycin resistance plasmid, pkd13, and primers ggatcgtctgctgacccgtgaatctttccatctcatgggtgtaggctggagctgcttc and gtctgaatataacgcggatgcgccggatcttcttccacattccggggatccgtcgacc. Cultures were grown in LB to OD_600_ = 0.6, fixed with 2∶1 volumes of RNAprotect Bacteria (Qiagen) and harvested. RNA was isolated from the pellet using SV RNA isolation kit (Promega) according to manufacturers instructions. RNA quality was determined using Bioanalyser (Agilent) and quantified using the ND-1000 (NanoDrop Technologies) after every manipulation step. 23S and 16S rRNA were depleted using MicrobExpress kit (Ambion). Genomic DNA was removed with two digestions using Amplification grade DNAse 1 (Invitrogen) to below PCR-detectable levels. The effect of incomplete DNAse treatment was a general increase in background (Figure S6). RNA was reverse transcribed using random primers (Invitrogen) and Superscript III (Invitrogen) at 45 C for three hours and heat denatured at 70 C for 15 minutes. Second strand synthesis was omitted in order to retain strand specific sequence determination; validation of this method is presented in full elsewhere (Croucher N, Fookes M, Perkins T. et al, submitted). Highly transcribed genes *fliC*, *tviB* and *vexA*, with a maximum amplicon of 250 bp, were used as targets for a PCR as a positive control for reverse transcription.

### Library construction and sequencing

Sequencing libraries for the Illumina GA platform were constructed by shearing the enriched cDNA by nebulisation (35psi, 6 min) followed by end-repair with Klenow polymerase, T4 DNA polymerase and T4 polynucleotide kinase (to blunt-end the DNA fragments). A single 3′ adenosine moiety was added to the cDNA using Klenow exo- and dATP. The Illumina adapters (containing primer sites for sequencing and flowcell surface annealing) were ligated onto the repaired ends on the cDNA and gel-electrophoresis was used to separate library DNA fragments from unligated adapters by selecting cDNA fragments between 200–250 bps in size. Fragmentation followed by gel-electrophoresis were used to separate library DNA fragments and size fragments were recovered following gel extraction at room temperature to ensure representation of AT rich sequences. Ligated cDNA fragments were recovered following gel extraction at room temperature to ensure representation of AT rich sequences. Libraries were amplified by 18 cycles of PCR with Phusion polymerase. Sequencing libraries were denatured with sodium hydroxide and diluted to 3.5 pM in hybridisation buffer for loading onto a single lane of an Illumina GA flowcell. Cluster formation, primer hybridisation and single-end, 36 cycle sequencing were performed using proprietary reagents according to manufacturers' recommended protocol (https://icom.illumina.com/). The efficacy of each stage of library construction was ascertained in a quality control step that involved measuring the adapter-cDNA on a Agilent DNA 1000 chip. A final dilution of 2 nM of the library was loaded onto the sequencing machine.

### Read mapping and visualization

We used the computational pipeline developed at the Wellcome Trust Sanger Institute, (http://www.sanger.ac.uk/Projects/Pathogens/Transcriptome/). We mapped all reads to the *S.* Typhi Ty2 genome using MAQ and discarded all reads that did not align uniquely to the genome. The quality parameter (−q) used in MAQ pileup was 30. MAQ pileup prints an array of delimited information formatted as one line per genomic base. Each base is assigned a value for the number of piled sequences and the mapped strand for each read, represented by a “.” (forward) and “,” (reverse). For example, Forward strand: all_bases, 7887, G, 45, @.............................................; Reverse strand: all_bases, 914, G, 6, @,,,,,,,; Overlapping Strands: all_bases, 7690, G, 38, @,,,,,.,.,,..,,,,,,.................... These data were then mapped strand specifically using the perl script maqpileup2depth.pl returning a plot file with two columns which can be read into Artemis as a graph by using commands “Graph, Add User Plot”.

### Secondary structure and conservation analyses for *S.* Typhi non-coding candidates

Candidate ncRNA sequences from Salmonella enterica subsp. enterica serovar Typhi Ty2 complete genome (EMBL ACC: AE014613.1) were searched against RFAMSEQ (a subset of the EMBL nucleotide database) using the Rfam search pipeline based upon WU-BLAST filters followed by covariance model (CM) scoring [13]. CMs have been proven to be vastly more accurate than BLAST for scoring ncRNAs [53]. Reliable matches were subsequently aligned and a consensus RNA secondary structure predicted folded using WAR [54]. Covariance models (CMs) were built for each resulting alignment; these researched searched against RFAMSEQ using the Rfam pipeline until there were no new reliable hits [13]. The subsequent alignments and secondary structures were inspected and modified by hand where improvements could be made. The secondary structure diagrams [55] and phylogenetic trees were built from these results. The alignments were then screened with the RNAz suite of tools for de-novo ncRNA prediction tool [23]. The original candidate sequences from *S.* typhi Ty2 were also analysed for individual secondary structure content using a permutation test. One thousand shuffled sequences with the same di-nucleotide content were generated for each native sequence. The distribution of predicted minimum free-energy (MFE) values of folding for the shuffled ensembl of sequences was used to determine the significance of the MFE value for the native sequence. There is an extensive literature on this approach with mixed success, the method is best suited to highly stable structures such as microRNAs [56],[57],[58].

### Comparative analysis of ssRNA–seq and microarray data

AM per base pair was determined using the script tram.pl and this value used as an expression value like fluorescence intensity on a microarray. The data from both microarray and ssRNA-seq were quantile normalised and differential analysis performed using the LIMMA package [41].

### Microarray scanning, hybridisation

We isolated RNA from three biological replicates and for each, four slides were hybridised using 16 µg of RNA and compared to the same amount of BRD948 RNA. The dyes were swapped for two arrays in each replicate. Low density spotted microarrays were used. Design, hybridisation and scanning were performed as previously described in Doyle *et al* [59] and array data submitted to Array Express. Overall 216 genes were identified as being differentially transcribed (2-fold, adj p-value<0.05) and 73 of these were reduced in transcription compared with BRD948.

### Cellular fractionation and protein sequencing

Whole cells were fractionated as previously described by Hantke [60]. Protein samples were reduced and alkylated with iodoacetamide prior a separation in a 4–12% NuPAGE Bis-Tris gel (Invitrogen). Gels were stained with colloidal Coomassie blue (Sigma) and bands were excised and followed by in-gel digestion by trypsin (sequencing grade; Roche). The extracted peptides were analyzed with on-line nano LC-MS/MS on an Ultimate 3000 Nano/Capillary LC System (Dionex) coupled to a LTQ FT Ultra mass spectrometer (ThermoElectron) equipped with a nanoelectrospray ion source (NSI). Samples were first loaded and desalted on a PepMap C18 trap (0.3 mm id×5 mm, Dionex) then separated on a BEH C18 analytical column (75 µm id×10 cm) [7] over a 30 or 45 or 60 min linear gradient of 4–32% CH_3_CN/0.1% FA based on the gel band's size and intensity. The mass spectrometer was operated in the standard data dependent acquisition mode controlled by Xcalibur 2.0. The survey scans (m/z 400–1500) were acquired on the FT-ICR at a resolution of 100,000 at m/z and the three most abundant multiply-charged ions (2+ and 3+) with a minimal intensity at 1000 counts were subject to MS/MS in the linear ion trap. The dynamic exclusion width was set at ±10 ppm. The automatic gain control (AGC) target value and maximum injection time were set at 1×10^6^ and 1000 msec for FT and 1×10^4^ and 250 msec for ion trap respectively. The instrument was externally calibrated. The Raw files were processed by BioWorks 3.3 and then submitted to a database search in Mascot server 2.2 (www.MatrixScience.com) against an in-house built Typhi Ty2 genomic 6-frame translated database [61]. All peptides with a posterior error probability (probability that an individual peptide was identified by chance alone) of 1% or less were accepted for subsequent analysis, resulting in an overall false discovery rate of about 0.1%. The analysed proteomic data has been submitted to EBI PRIDE database (www.ebi.ac.uk/pride/) with and can be viwed under PRIDE accession number 9770–9774.

### Peptide mapping script

The peptide sequences were mapped to all matching positions in a 6-frame translation of the entire genome and only peptides that mapped to one region of the genome were included in these data.

## Supporting Information

Figure S1 Paralogues of putative ncRNA identified in this study. AM for paralogues (mean and range) of (a) RUF_107c and (b) RUF_175c.
(0.29 MB PDF)

Figure S2 Predicted secondary structure of transcript mapping to (a) RUF_220c, the upstream region of *sprA*, (b) RUF_219c, the upstream region of *sprB* and (c) RUF_221, the upstream region of *iagA*.
(0.81 MB PDF)

Figure S3 AM values for pseudogenes with respect to predicted age. Eldest pseudogenes, left and most recent, right.
(0.25 MB PDF)

Figure S4 Genes differentially expressed (2-fold, p<0.05) in both the microarray data and Illumina generated data.
(0.28 MB PDF)

Figure S5 Threonine leader attenuation. Translation of the threonine rich leader peptide, ThrL, arrests transcription of the downstream threonine biosynthesis genes.
(0.22 MB PDF)

Figure S6 Impact of DNA contamination on ssRNA-seq. Artemis representation of ssRNA-seq data plots from S. Typhi Ty2. Uppermost plot A represents data from a sample that was digested by two rounds of DNAse 1 and passed quality control that are described in the methods. Lower most plot B represents data from a sample that was digested with only one round of DNAse 1 digestion and had detectable DNA contamination. Both datasets were mapped using the same parameters. ds-DNA preferentially ligates to linkers and absorbs sequencing capacity, which reduces the overally efficacy of ssRNA-seq. All plots that were used in this study were scanned for contaminating gDNA, which normally maps consistently across the genome whereas completely DNAse 1 digested samples contain regions of no mapped sequence data.
(0.07 MB PDF)

Table S1 Ty2 Annotated genes with at least one sequenced peptide mapped.
(0.02 MB XLS)

Table S2 Depth coverage of known non-coding RNAs and novel RUFs for each experiment.
(0.14 MB XLS)

Table S3 Genes differentially expressed in DNA microarray experiments.
(0.05 MB PDF)

Table S4 Ty2 genes annotated as phage genes with uniquely mapped sequenced peptides.
(0.04 MB XLS)
