# Supplementary figures and images for: A Strand-Specific RNA–Seq Analysis of the Transcriptome of the Typhoid Bacillus Salmonella Typhi
Source: PLoS Genet. 2009 Jul 17;5(7):e1000569. doi: 10.1371/journal.pgen.1000569 (PMC2704369; doi:10.1371/journal.pgen.1000569)

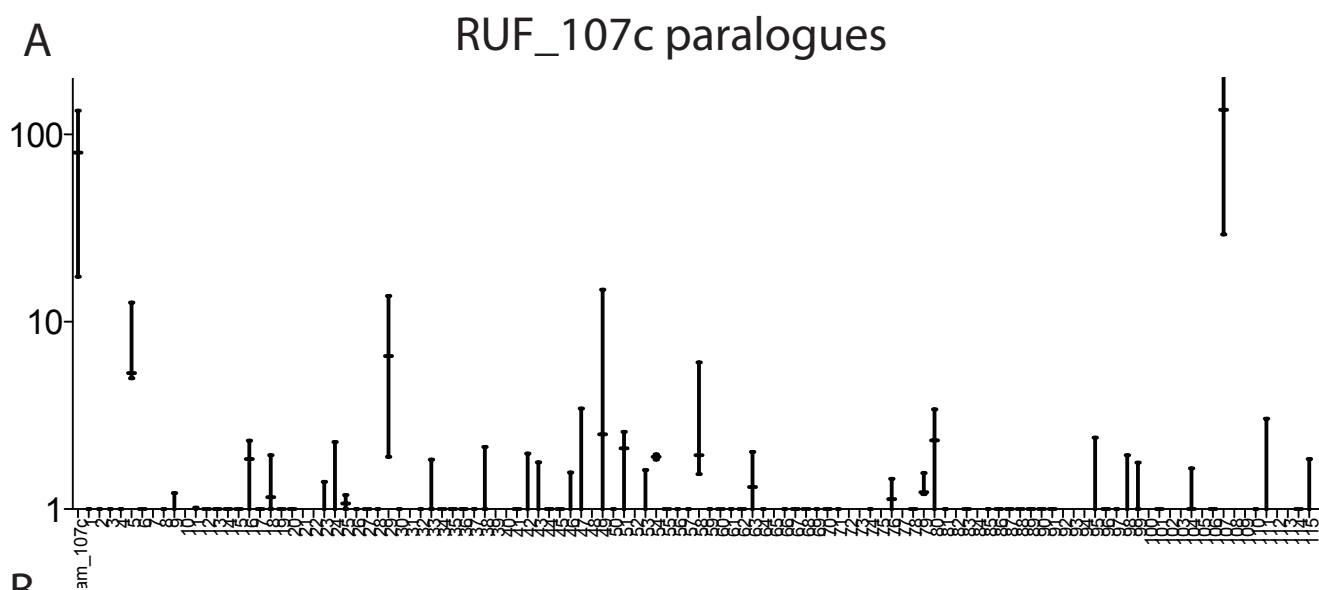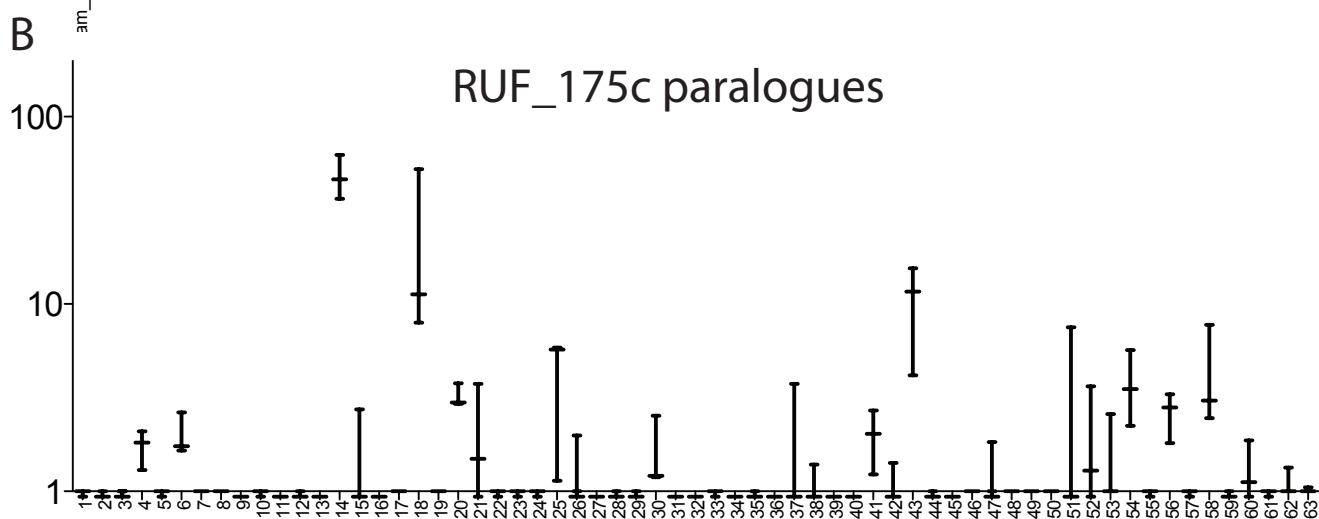

Supplement: Figure S1 — Paralogues of putative ncRNA identified in this study. AM for paralogues (mean and range) of (a) RUF_107c and (b) RUF_175c. (0.29 MB PDF) [file pgen.1000569.s001.pdf]

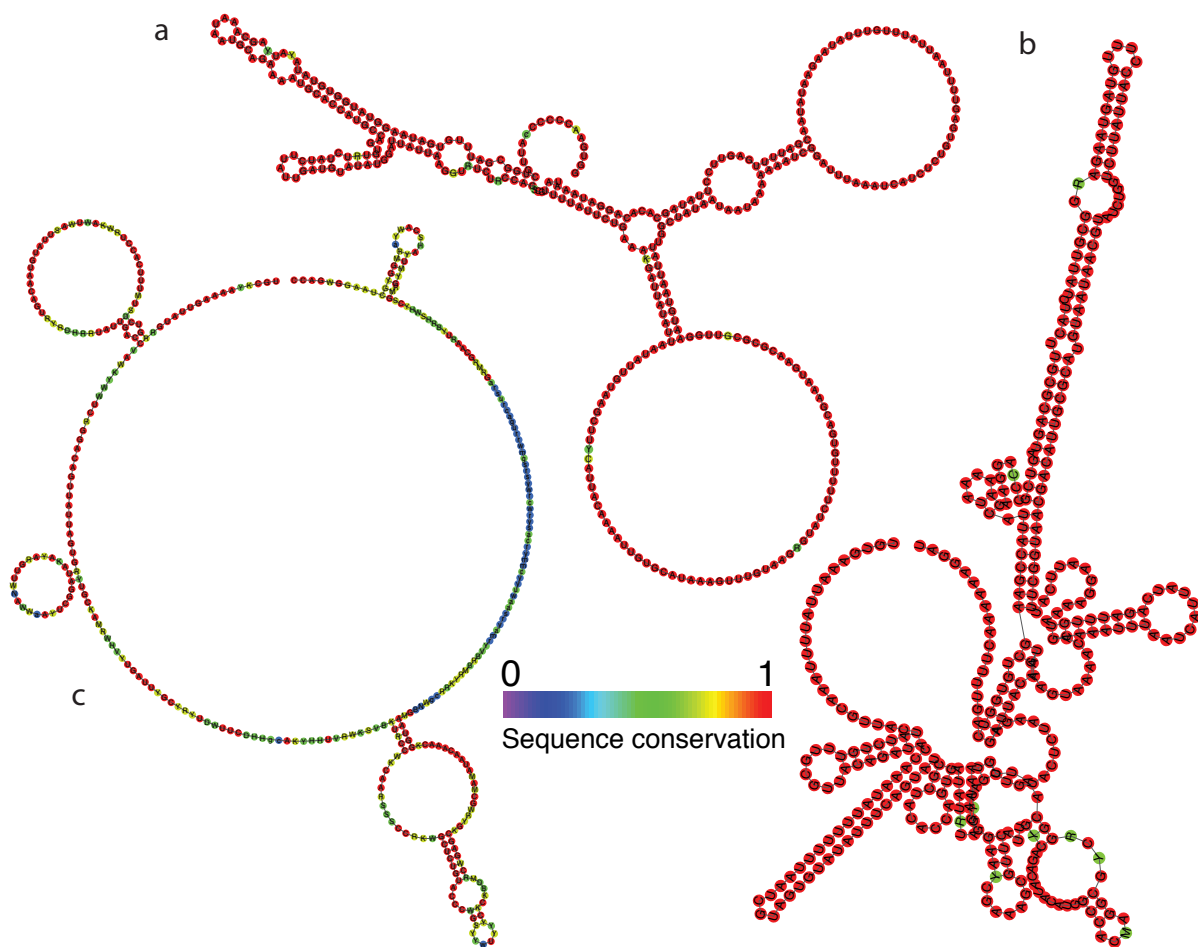

Supplement: Figure S2 — Predicted secondary structure of transcript mapping to (a) RUF_220c, the upstream region of sprA, (b) RUF_219c, the upstream region of sprB and (c) RUF_221, the upstream region of iagA. (0.81 MB PDF) [file pgen.1000569.s002.pdf]

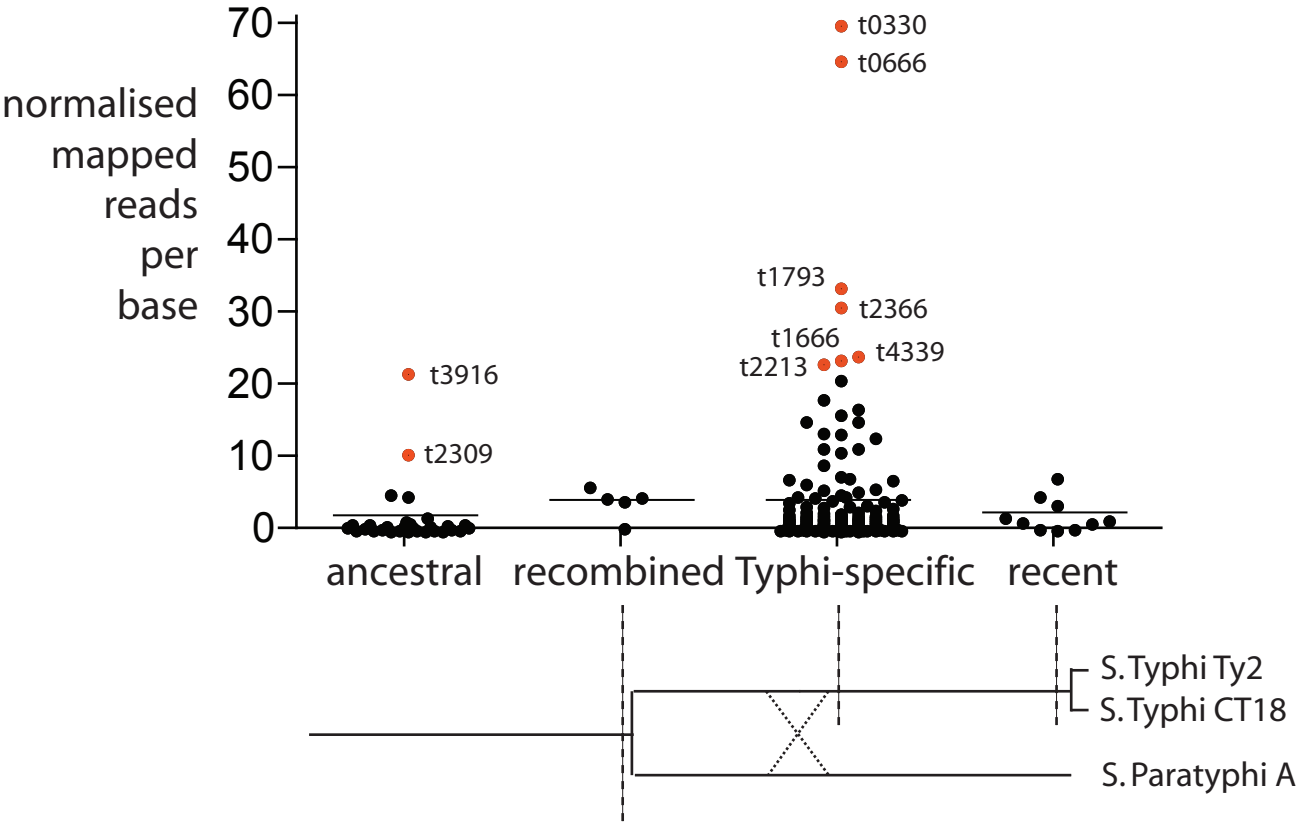

Supplement: Figure S3 — AM values for pseudogenes with respect to predicted age. Eldest pseudogenes, left and most recent, right. (0.25 MB PDF) [file pgen.1000569.s003.pdf]

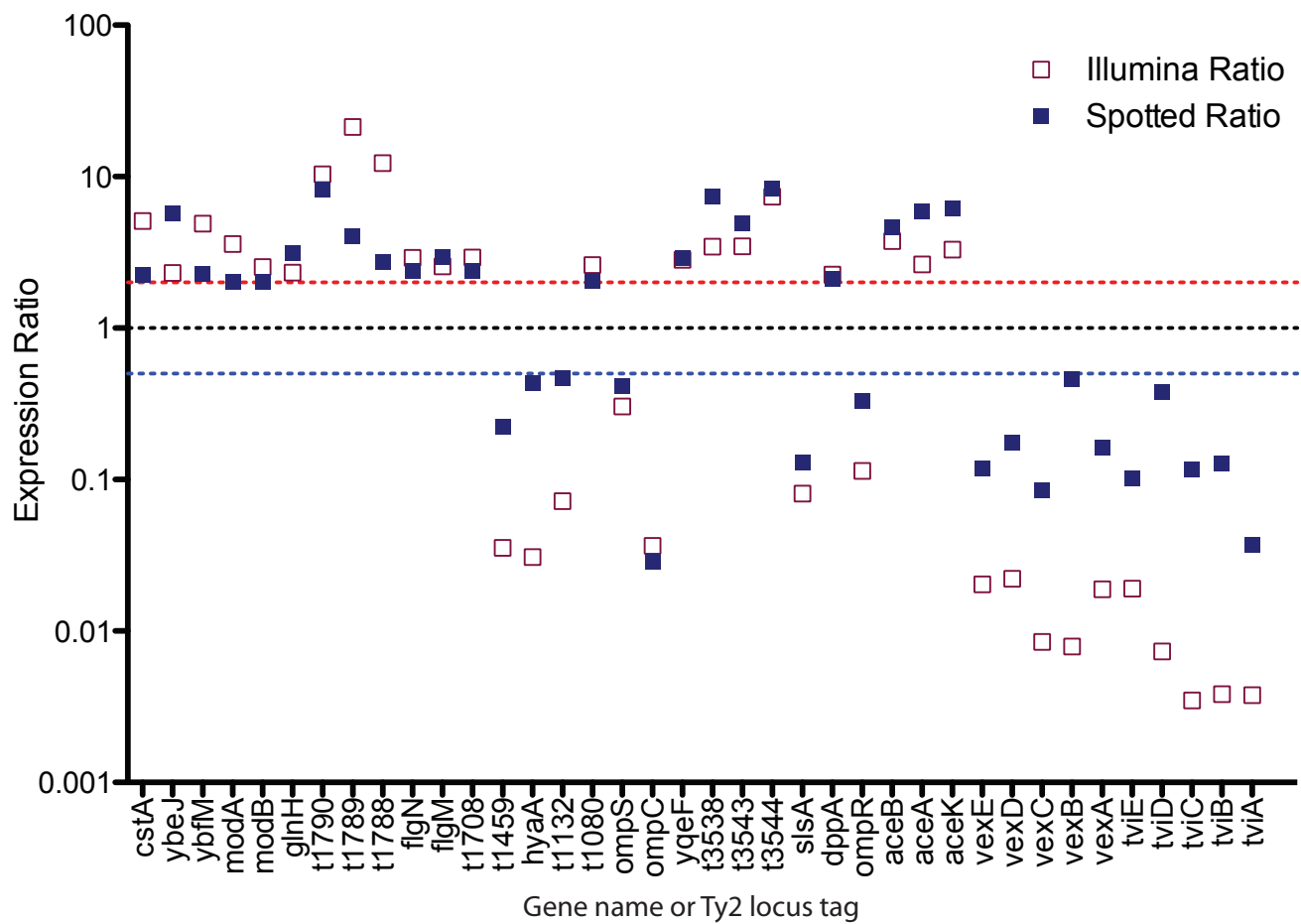

Supplementary figure 4

Supplement: Figure S4 — Genes differentially expressed (2-fold, p<0.05) in both the microarray data and Illumina generated data. (0.28 MB PDF) [file pgen.1000569.s004.pdf]

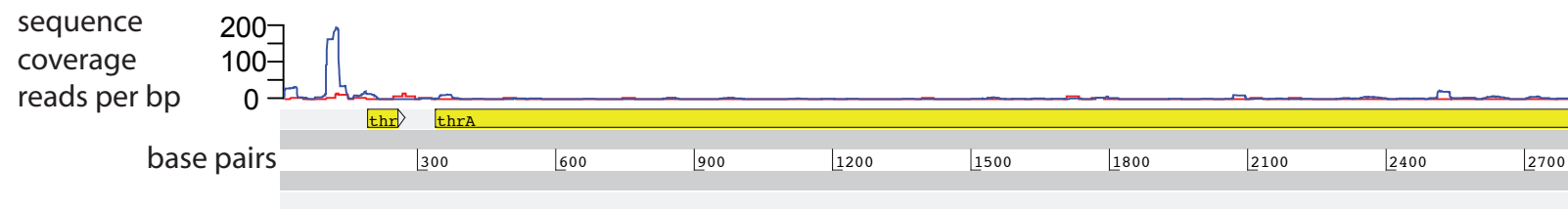

Supplementary figure 5

Supplement: Figure S5 — Threonine leader attenuation. Translation of the threonine rich leader peptide, ThrL, arrests transcription of the downstream threonine biosynthesis genes. (0.22 MB PDF) [file pgen.1000569.s005.pdf]

User algorithm from 1104\_s\_2.plot Window size: 50

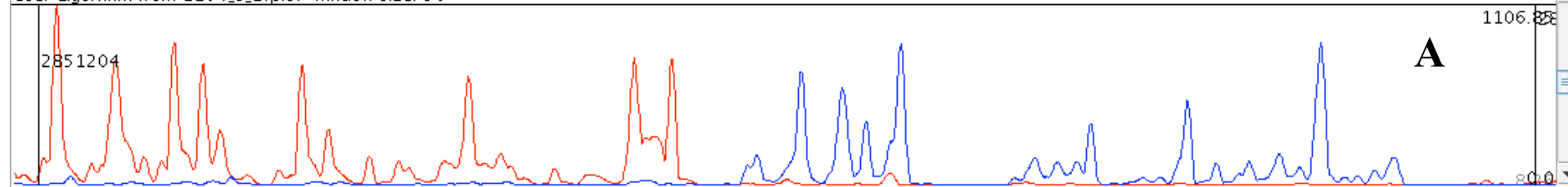

User algorithm from 876\_s\_3.plot Window size: 50

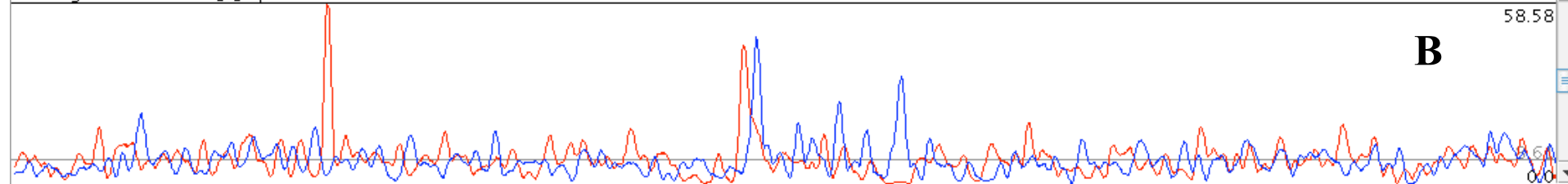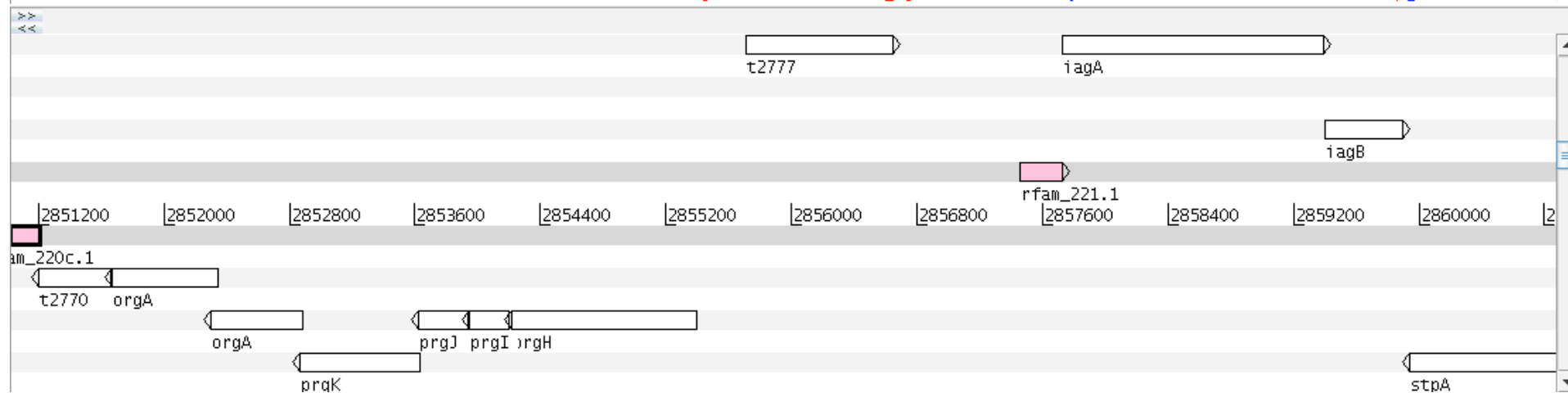

Supplement: Figure S6 — Impact of DNA contamination on ssRNA-seq. Artemis representation of ssRNA-seq data plots from S. Typhi Ty2. Uppermost plot A represents data from a sample that was digested by two rounds of DNAse 1 and passed quality control that are described in the methods. Lower most plot B represents data from a sample that was digested with only one round of DNAse 1 digestion and had detectable DNA contamination. Both datasets were mapped using the same parameters. ds-DNA preferentially ligates to linkers and absorbs sequencing capacity, which reduces the overally efficacy of ssRNA-seq. All plots that were used in this study were scanned for contaminating gDNA, which normally maps consistently across the genome whereas completely DNAse 1 digested samples contain regions of no mapped sequence data. (0.07 MB PDF) [file pgen.1000569.s006.pdf]
