# Supplementary material for: A Strand-Specific RNA–Seq Analysis of the Transcriptome of the Typhoid Bacillus Salmonella Typhi
Source: PLoS Genet. 2009 Jul 17;5(7):e1000569. doi: 10.1371/journal.pgen.1000569 (PMC2704369; doi:10.1371/journal.pgen.1000569)
Supplement: Table S3 — Genes differentially expressed in DNA microarray experiments. (0.05 MB PDF) [file pgen.1000569.s009.pdf]

Locus Tag	Gene Symbol	CT18 Orthologue	Expression Ratio	t	P.Value	Product String	Accession
t0011	yaaI	STY0011	2.91	3.25	0.017	hypothetical protein	AL513382
t0030	NA	STY0034	0.45	-2.70	0.034	hypothetical protein	AL513382
t0065	citG	STY0072	2.38	3.16	0.018	CitG protein	AL513382
t0070	caiE	STY0079	2.57	2.93	0.025	carnitine operon protein CaiE	AL513382
t0083	NA	STY0095	2.04	3.88	0.008	probable secreted protein	AL513382
t0248	metQ	STY0272	2.41	2.49	0.046	DL-methionine transporter substrate-binding subunit	AL513382
t0310	suhB	STY2792	0.31	-2.72	0.033	inositol monophosphatase	AL513382
t0364	NA	STY2734	3.04	3.98	0.007	hypothetical protein	AL513382
t0375	NA	STY2722	2.38	3.47	0.012	hypothetical protein	AL513382
t0391	eutQ	STY2704	2.84	5.08	0.002	putative ethanolamine utilization protein EutQ	AL513382
t0408	amiA	STY2687	0.47	-2.65	0.037	N-acetylmuramoyl-l-alanine amidase I	AL513382
t0450	NA	STY2648	2.56	3.01	0.023	hypothetical protein	AL513382
t0523	NA	STY2571	3.17	3.66	0.010	putative transketolase N-terminal section	AL513382
t0569	NA	STY2524	0.18	-7.31	0.000	hypothetical protein	AL513382
t0575	NA	STY2518	0.43	-2.82	0.029	hypothetical protein	AL513382
t0618	narP	STY2472	0.37	-2.66	0.036	DNA-binding response regulator in two-component regulatory system with NarQ or NarX	AL513382
t0620	NA	STY2470	0.28	-3.90	0.007	homolog of virulence protein msgA	AL513382
t0646	NA	STY2445	0.08	-4.03	0.006	hypothetical protein	AL513382
t0656	cirA	STY2434	2.35	2.67	0.036	ferric iron-catecholate outer membrane transporter	AL513382
t0659	sdaC	STY2430	0.30	-3.14	0.019	putative L-serine dehydratase	AL513382
t0682	NA	STY2403	0.36	-2.99	0.023	hypothetical protein	AL513382
t0692	yehY	STY2394	3.16	4.32	0.005	putative permease transmembrane component	AL513382
t0704	stcA	STY2381	0.25	-2.53	0.043	putative fimbrial subunit protein	AL513382
t0707	stcD	STY2378	0.32	-3.44	0.013	hypothetical protein	AL513382
t0721	NA	STY2364	2.10	2.88	0.027	hypothetical protein	AL513382
t0763	gmd	STY2321	2.28	3.38	0.014	GDP-mannose 4,6-dehydratase	AL513382
t0803	hisL	STY2279A	0.31	-3.00	0.023	his operon leader peptide	AL513382

t0810	phsB	STY2270	2.22	2.84	0.028	thiosulfate reductase electron transport protein	AL513382
t0811	phsC	STY2269	2.07	2.66	0.036	thiosulfate reductase cytochrome b subunit	AL513382
t0828	pduK	STY2251	2.73	3.21	0.017	putative propanediol utilization protein PduK	AL513382
t0837	pduF	STY2242	2.04	3.05	0.021	propanediol diffusion facilitator	AL513382
t0883	ompS	STY2203	0.30	-3.85	0.008	outer membrane protein S1	AL513382
t0890	NA	STY2195	0.49	-2.60	0.040	hypothetical protein	AL513382
t0895	rcsA	STY2190	5.39	3.31	0.015	colanic acid capsullar biosynthesis activation protein A	AL513382
t0913	yedD	STY2172	2.43	3.44	0.013	hypothetical protein	AL513382
t0922	fliY	STY2162	3.28	4.78	0.003	cystine transporter subunit	AL513382
t0961	cheY	STY2125	2.20	2.65	0.037	chemotaxis regulator transmitting signal to flagellar motor component	AL513382
t0965	flhE	STY2121	3.98	4.67	0.003	flagellar protein FlhE precursor	AL513382
t0975	yecD	STY2110	0.14	-3.69	0.010	hypothetical protein	AL513382
t1011	NA	STY2005	2.18	2.52	0.044	hypothetical protein	AL513382
t1012	yjcS	STY2004	2.05	3.37	0.014	putative hydrolase	AL513382
t1022	NA	STY1988	0.08	-3.98	0.007	hypothetical protein	AL513382
t1037	NA	STY1971	3.57	2.89	0.027	hypothetical protein	AL513382
t1042	ftsI	STY1965	0.49	-2.96	0.024	penicillin-binding protein	AL513382
t1045	NA	STY1962	0.36	-2.99	0.023	hypothetical protein	AL513382
t1050	NA	STY1957	2.27	3.57	0.011	hypothetical protein	AL513382
t1073	NA	STY1933	2.03	2.53	0.043	hypothetical protein	AL513382
t1080	NA	STY1925	2.60	2.90	0.026	hypothetical protein	AL513382
t1094	NA	STY1908	3.10	3.79	0.008	predicted inner membrane protein	AL513382
t1107	NA	STY1891	0.30	-2.52	0.044	putative pertussis-like toxin subunit	AL513382
t1116	pagD	STY1880A	0.31	-2.51	0.044	putative outer membrane virulence protein	AL513382
t1132	NA	STY1867	0.07	-2.62	0.038	putative lipoprotein	AL513382
t1141	NA	STY1858	4.82	2.88	0.027	hypothetical protein	AL513382
t1149	NA	STY1850	3.23	3.14	0.019	hypothetical protein	AL513382
t1190	osmE	STY1802	2.95	2.94	0.025	DNA-binding transcriptional activator	AL513382
t1195	celF	STY1797	2.27	2.48	0.047	phospho-beta-glucosidase B	AL513382
t1236	NA	STY1755	0.26	-2.72	0.033	putative transporter	AL513382

t1245	lppA	STY1745	0.22	-3.57	0.011	major outer membrane lipoprotein	AL513382
t1254	ttrS	STY1735	2.95	2.61	0.039	hypothetical protein	AL513382
t1255	ttrR	STY1733	2.51	3.06	0.021	putative two-component response regulator	AL513382
t1256	ydhZ	STY1732	3.40	2.79	0.030	hypothetical protein	AL513382
t1259	ssrB	STY1729	0.32	-2.60	0.039	putative two-component response regulator	AL513382
t1260	ssrA	STY1728	0.10	-5.32	0.002	putative two-component sensor kinase	AL513382
t1267	sscA	STY1721	2.39	2.98	0.023	putative type III secretion system chaperone protein	AL513382
t1276	ssaI	STY1712	0.27	-2.45	0.049	putative pathogenicity island protein	AL513382
t1281	ssaM	STY1707	2.07	3.79	0.009	putative pathogenicity island protein	AL513382
t1341	NA	STY1649	0.13	-5.47	0.001	outer membrane protein	AL513382
t1360	NA	STY1628	0.34	-3.73	0.009	hypothetical protein	AL513382
t1364	NA	STY1624	0.24	-6.97	0.000	hypothetical protein	AL513382
t1366	NA	STY1622	2.82	3.56	0.011	conserved bacteriophage hypothetical protein	AL513382
t1397	NA	STY1591	0.30	-3.84	0.008	putative bacteriophage transcriptional regulator	AL513382
t1413	NA	STY1572	2.62	2.63	0.038	putative ABC transporter membrane protein	AL513382
t1414	NA	STY1571	2.57	4.26	0.005	putative ABC transporter periplasmic binding protein	AL513382
t1430	NA	STY1552	2.31	2.81	0.030	hypothetical protein	AL513382
t1451	NA	STY1531	0.20	-6.46	0.001	putative ATP/GTP-binding protein	AL513382
t1458	hyaA	STY1523	0.03	-14.36	0.000	uptake hydrogenase small subunit	AL513382
t1459	NA	STY1522	0.04	-12.29	0.000	putative secreted hydrolase	AL513382
t1470	NA	STY1507	2.82	2.76	0.032	putative aminotransferase	AL513382
t1474	NA	STY1502	2.90	2.78	0.031	putative secreted protein	AL513382
t1488	narZ	STY1488	0.42	-3.91	0.007	respiratory nitrate reductase 2 alpha chain	AL513382
t1489	narY	STY1487	0.38	-4.26	0.005	respiratory nitrate reductase 2 beta chain	AL513382
t1535	NA	STY1439	0.46	-2.60	0.039	hypothetical protein	AL513382

t1553	NA	STY1419	0.36	-3.52	0.012	probable pyruvate-flavodoxin oxidoreductase	AL513382
t1561	NA	STY1408	2.51	5.27	0.002	putative chemo-receptor protein	AL513382
t1562	NA	STY1406	2.03	2.84	0.028	hypothetical protein	AL513382
t1566	NA	STY1402	0.47	-3.04	0.022	hypothetical protein	AL513382
t1568	NA	STY1400	2.22	3.57	0.011	conserved hypothetical DNA-binding protein	AL513382
t1581	NA	STY1386	2.90	3.88	0.008	putative transcriptional regulator	AL513382
t1640	NA	STY1323	0.25	-3.24	0.017	hypothetical protein	AL513382
t1641	NA	STY1322	0.10	-11.50	0.000	hypothetical protein	AL513382
t1642	NA	STY1321	0.05	-5.67	0.001	hypothetical protein	AL513382
t1643	NA	STY1320	0.23	-4.33	0.005	hypothetical protein	AL513382
t1708	NA	STY1252	2.93	4.35	0.004	hypothetical protein	AL513382
t1749	flgN	STY1210	2.90	2.67	0.036	flagella synthesis protein FlgN	AL513382
t1753	rimJ	STY1205	2.10	3.54	0.011	ribosomal-protein-S5-alanine N-acetyltransferase	AL513382
t1773	NA	STY1184	2.41	2.71	0.034	hypothetical protein	AL513382
t1788	NA	STY1169	12.26	3.11	0.020	putative sialic acid transporter	AL513382
t1789	NA	STY1168	21.25	3.02	0.022	putative secreted protein	AL513382
t1790	NA	STY1167	10.37	2.77	0.031	hypothetical protein	AL513382
t1792	NA	STY1165	10.03	2.47	0.047	putative membrane transporter	AL513382
t1800	NA	STY1156	0.05	-7.65	0.000	hypothetical protein	AL513382
t1867	NA	STY2013	0.25	-3.20	0.018	putative bacteriophage tail protein	AL513382
t1870	NA	STY2016	0.31	-4.16	0.005	putative bacteriophage protein	AL513382
t1884	NA	STY2030	0.39	-3.32	0.015	putative bacteriophage protein	AL513382
t1892	NA	STY2038	0.34	-3.01	0.023	hypothetical protein	AL513382
t1912	NA	STY1028	3.71	2.98	0.024	putative bacteriophage protein	AL513382
t1916	NA	STY1024	0.39	-3.80	0.008	putative DNA-binding protein	AL513382
t1923	betA	STY1017	0.45	-2.66	0.036	bacteriophage recombination protein	AL513382
t1924	exo	STY1016	0.49	-2.97	0.024	exonuclease	AL513382
t1926	NA	STY1014	0.46	-2.97	0.024	putative DNA methylase	AL513382
t1959	NA	STY0975	0.40	-3.00	0.023	hypothetical protein	AL513382
t2016	potI	STY0913	2.86	3.14	0.019	putrescine transporter subunit: membrane component of ABC superfamily	AL513382
t2063	ybiI	STY0862	2.10	2.81	0.029	hypothetical protein	AL513382
t2077	ybhN	STY0846	2.40	2.78	0.031	hypothetical protein	AL513382

t2095	ybhB	STY0825	0.25	-3.48	0.012	predicted kinase inhibitor	AL513382
t2101	ybhC	STY0819	0.45	-3.64	0.010	possible pectinesterase precursor	AL513382
t2107	ybhT	STY0813	0.34	-3.38	0.014	hypothetical protein	AL513382
t2188	ybfM	STY0725	4.90	5.69	0.001	putative outer membrane protein	AL513382
t2208	ybeJ	STY0710	2.32	2.46	0.048	glutamate and aspartate transporter subunit	AL513382
t2252	ybdR	STY0663	2.64	4.99	0.002	hypothetical zinc-dependant alcohol dehydrogenase	AL513382
t2267	NA	STY0645	3.11	4.61	0.003	hypothetical protein	AL513382
t2268	cstA	STY0644	5.09	6.14	0.001	carbon starvation protein A	AL513382
t2303	gtrA	STY0607	0.45	-3.03	0.022	bactoprenol-linked glucose translocase	AL513382
t2336	glxK	STY0573	2.47	3.65	0.010	glycerate kinase II	AL513382
t2350	sfbA	STY0558	4.65	6.23	0.001	lipoprotein	AL513382
t2380	ybaM	STY0524	2.53	2.72	0.033	hypothetical protein	AL513382
t2401	NA	STY0501	2.71	3.97	0.007	putative lyase	AL513382
t2425	NA	STY0477	3.49	4.29	0.005	hypothetical protein	AL513382
t2436	phnU	STY0466	2.93	4.40	0.004	probable membrane component of 2-aminoethylphosphonate transporter	AL513382
t2437	phnV	STY0465	2.43	3.56	0.011	probable membrane component of 2-aminoethylphosphonate transporter	AL513382
t2450	tsx	STY0451	3.73	2.77	0.031	nucleoside-specific channel-forming protein tsx precursor	AL513382
t2452	yajD	STY0449	2.11	2.79	0.030	hypothetical protein	AL513382
t2490	NA	STY0406	0.47	-3.75	0.009	putative DNA-binding transcriptional regulator	AL513382
t2508	NA	STY0387	0.21	-7.32	0.000	putative metabolite transport protein	AL513382
t2543	NA	STY0352	0.12	-6.39	0.001	probable secreted protein	AL513382
t2567	NA	STY0323	0.46	-3.36	0.014	hypothetical protein	AL513382
t2598	NA	STY0287	2.62	2.59	0.040	hypothetical protein	AL513382
t2734	srlE	STY2954	3.02	2.52	0.044	glucitol/sorbitol-specific IIBC component of PTS system	AL513382
t2748	hycH	STY2968	2.21	2.45	0.049	formate hydrogenlyase maturation protein	AL513382
t2749	hycG	STY2969	2.65	2.46	0.047	formate hydrogenlyase subunit 7	AL513382
t2809	NA	STY3032	0.17	-3.22	0.017	hypothetical protein	AL513382
t2833	ygbE	STY3057	0.45	-2.55	0.042	hypothetical protein	AL513382

t2851	NA	STY3078	2.51	2.89	0.026	hypothetical protein	AL513382
t2940	stdA	STY3177	0.22	-2.69	0.035	probable fimbrial protein	AL513382
t2994	NA	STY3233	2.60	2.44	0.049	possible ABC-transport protein, ATP-binding component	AL513382
t3014	NA	STY3255	0.49	-4.03	0.006	hypothetical protein	AL513382
t3026	nupG	STY3268	2.42	4.27	0.005	nucleoside permease	AL513382
t3033	NA	STY3278	0.27	-2.99	0.023	hypothetical protein	AL513382
t3074	NA	STY3326	3.22	2.92	0.025	hypothetical protein	AL513382
t3142	ygjQ	STY3402	2.11	2.57	0.041	hypothetical protein	AL513382
t3147	NA	STY3407	2.23	3.85	0.008	hypothetical protein	AL513382
t3151	NA	STY3411	2.03	3.75	0.009	hypothetical protein	AL513382
t3194	NA	STY3457	0.18	-4.23	0.005	putative protease	AL513382
t3219	NA	STY3481	0.44	-2.73	0.033	hypothetical protein	AL513382
t3221	rplU	STY3483	0.36	-5.02	0.002	50S ribosomal protein L21	AL513382
t3276	NA	STY3542	0.19	-3.27	0.016	hypothetical protein	AL513382
t3342	NA	STY3604	2.06	3.15	0.019	chloramphenicol-sensitive protein RarD	AL513382
t3356	NA	STY3618	0.15	-2.75	0.032	hypothetical protein	AL513382
t3404	cII	STY3662	0.44	-2.43	0.050	regulatory protein cII	AL513382
t3408	NA	STY3666	0.27	-2.71	0.034	hypothetical protein	AL513382
t3543	NA	STY3795	3.46	2.46	0.048	putative ABC transporter permease protein	AL513382
t3544	NA	STY3796	7.37	3.16	0.019	putative ABC transporter ATP-binding protein	AL513382
t3619	NA	STY3879	3.02	2.64	0.037	hypothetical protein	AL513382
t3638	rbsD	STY3897	0.20	-4.33	0.004	high affinity ribose transport protein RbsD	AL513382
t3660	stgB	STY3919	0.39	-3.09	0.020	fimbrial chaperone protein	AL513382
t3691	NA	STY3950	2.27	3.81	0.008	hypothetical protein	AL513382
t3728	uhpA	STY3992	2.19	2.75	0.032	DNA-binding response regulator in two-component regulatory system wtih UhpB	AL513382
t3750	NA	STY4017	2.05	2.62	0.038	putative transferase	AL513382
t3757	slsA	STY4025	0.08	-8.42	0.000	hypothetical protein	AL513382
t3806	waaL	STY4082	0.46	-2.57	0.041	O-antigen ligase	AL513382
t3841	NA	STY4118	2.72	3.26	0.016	putative transcriptional regulator	AL513382
t3885	dppA	STY4168	2.25	3.86	0.008	periplasmic dipeptide transport protein precursor	AL513382

t3912	NA	STY4198	0.28	-3.79	0.008	phage-like lysozyme	AL513382
t3973	NA	STY4263	0.29	-2.53	0.043	hypothetical protein	AL513382
t4017	yrfA	STY4307	5.76	4.13	0.006	hypothetical protein	AL513382
t4033	ppiA	STY4324	0.35	-3.42	0.013	peptidyl-prolyl cis-trans isomerase A (rotamase A)	AL513382
t4100	smg	STY4393	2.48	4.39	0.004	hypothetical protein	AL513382
t4128	NA	STY4418	0.47	-3.51	0.012	hypothetical protein	AL513382
t4180	NA	STY4472	2.95	4.02	0.006	hypothetical protein	AL513382
t4200	NA	STY4492	0.45	-3.42	0.013	predicted metal dependent hydrolase	AL513382
t4201	NA	STY4493	2.41	2.76	0.031	arginine:agmatin antiporter	AL513382
t4267	NA	STY4571	0.44	-2.72	0.033	putative lipoprotein	AL513382
t4271	NA	STY4574	2.20	3.45	0.013	hypothetical protein	AL513382
t4275	NA	STY4578	2.14	2.63	0.038	hypothetical protein	AL513382
t4289	NA	STY4595	2.29	4.24	0.005	hypothetical protein	AL513382
t4298	NA	STY4604	0.23	-4.30	0.005	hypothetical protein	AL513382
t4308	NA	STY4614	2.39	3.00	0.023	phage baseplate assembly protein	AL513382
t4326	NA	STY4632	4.23	2.43	0.049	hypothetical protein	AL513382
t4344	vexE	STY4651	0.02	-14.96	0.000	Vi polysaccharide export protein	AL513382
t4345	vexD	STY4652	0.02	-11.28	0.000	Vi polysaccharide export inner-membrane protein	AL513382
t4346	vexC	STY4653	0.01	-9.52	0.000	Vi polysaccharide export ATP-binding protein	AL513382
t4348	vexA	STY4655	0.02	-14.28	0.000	Vi polysaccharide export protein	AL513382
t4349	tviE	STY4656	0.02	-14.07	0.000	Vi polysaccharide biosynthesis protein TviE	AL513382
t4350	tviD	STY4659	0.01	-16.64	0.000	Vi polysaccharide biosynthesis protein	AL513382
t4351	tviC	STY4660	0.00	-14.41	0.000	Vi polysaccharide biosynthesis protein, epimerase	AL513382
t4352	tviB	STY4661	0.00	-13.21	0.000	Vi polysaccharide biosynthesis protein, UDP- glucose/GDP-mannose dehydrogenase	AL513382
t4353	tviA	STY4662	0.00	-8.72	0.000	Vi polysaccharide biosynthesis protein	AL513382
t4354	NA	STY4663	0.20	-2.99	0.023	hypothetical protein	AL513382
t4357	NA	STY4666	0.44	-3.79	0.008	probable phage integrase	AL513382
t4397	yjeM	STY4705	0.21	-5.04	0.002	putative amino acid permease	AL513382

t4398	yjeN	STY4706	0.26	-2.43	0.050	hypothetical protein	AL513382
t4466	NA	STY4771	2.05	2.71	0.034	putative sugar transporter	AL513382
t4475	NA	STY4780	3.69	4.63	0.003	hypothetical protein	AL513382
t4526	cI	STY4829	0.48	-2.90	0.026	phage immunity repressor protein	AL513382
t4593	NA	STY4903	2.20	2.46	0.048	ferric iron reductase involved in ferric hydroximate transport	AL513382
t4604	osmY	STY4911	0.35	-3.24	0.017	periplasmic protein	AL513382
